# Spatial cognition, body representation and affective processes: the role of vestibular information beyond ocular reflexes and control of posture

**DOI:** 10.3389/fnint.2014.00044

**Published:** 2014-05-27

**Authors:** Fred W. Mast, Nora Preuss, Matthias Hartmann, Luzia Grabherr

**Affiliations:** ^1^Department of Psychology, University of BernBern, Switzerland; ^2^Center for Cognition, Learning and Memory, University of BernBern, Switzerland; ^3^Sansom Institute for Health Research, University of South AustraliaAdelaide, SA, Australia

**Keywords:** vestibular system, vestibular imagery, multisensory integration, mental rotation, body representation, body schema, affect, mood

## Abstract

A growing number of studies in humans demonstrate the involvement of vestibular information in tasks that are seemingly remote from well-known functions such as space constancy or postural control. In this review article we point out three emerging streams of research highlighting the importance of vestibular input: (1) *Spatial Cognition*: Modulation of vestibular signals can induce specific changes in spatial cognitive tasks like mental imagery and the processing of numbers. This has been shown in studies manipulating body orientation (changing the input from the otoliths), body rotation (changing the input from the semicircular canals), in clinical findings with vestibular patients, and in studies carried out in microgravity. There is also an effect in the reverse direction; top-down processes can affect perception of vestibular stimuli. (2) *Body Representation*: Numerous studies demonstrate that vestibular stimulation changes the representation of body parts, and sensitivity to tactile input or pain. Thus, the vestibular system plays an integral role in multisensory coordination of body representation. (3) *Affective Processes and Disorders*: Studies in psychiatric patients and patients with a vestibular disorder report a high comorbidity of vestibular dysfunctions and psychiatric symptoms. Recent studies investigated the beneficial effect of vestibular stimulation on psychiatric disorders, and how vestibular input can change mood and affect. These three emerging streams of research in vestibular science are—at least in part—associated with different neuronal core mechanisms. Spatial transformations draw on parietal areas, body representation is associated with somatosensory areas, and affective processes involve insular and cingulate cortices, all of which receive vestibular input. Even though a wide range of different vestibular cortical projection areas has been ascertained, their functionality still is scarcely understood.

## Introduction

The wide majority of vestibular research concerns basic oculomotor mechanisms and postural control. Over several decades a substantial body of research has been ascertained, including the perception of self-motion and neuronal circuits that underlie the vestibulo-ocular reflex (VOR). The nature of the VOR has inspired computational models with precisely defined input parameters, and single-cell recordings have provided first insights into the brain areas that are involved in the processing of vestibular input (Grüsser et al., 1990). With the advent of neuroimaging, researchers started to use ways to induce vestibular sensations while brain activation was assessed. This is a challenging procedure because most current neuroimaging techniques require a stable head position, and it is therefore not possible to investigate the neuronal correlates of vestibular stimuli as they occur during natural head movements. However, the use of caloric vestibular stimulation (CVS), galvanic vestibular stimulation (GVS), and high-frequency auditory stimuli (clicks or tone bursts) allow for inducing vestibular stimulation while the head remains stationary. This research provided important findings about the human cortical areas associated with the processing of vestibular stimulation. The exact locations and functions of vestibular cortical areas are still a matter of debate (Lopez and Blanke, 2011). A recent meta-analysis by Lopez et al. (2012b) could identify the core regions of the human vestibular cortex. These areas involve the Sylvian fissure, insula, retroinsular cortex, fronto-parietal operculum, and cingulate cortex. Despite some discrepancies between different studies about the cortical areas involved in vestibular stimulation (Lopez et al., 2012b; zu Eulenburg et al., 2012) there is absolutely no doubt about the existence of widespread cortical vestibular representations.

Interestingly, there is still a lack of knowledge about the functions these cortical vestibular networks are involved in and with what other networks they overlap. For example, it has been shown that neurons in the gaze-related frontal eye fields are modulated by rotation and translation (Ebata et al., 2004). In middle superior temporal area (MSTd), neurons respond to inertial motion in darkness. These neurons represent visual-vestibular integration for heading perception (Gu et al., 2007). Neurons in the ventral intraparietal area (VIP) respond to passive translations (Schlack et al., 2002) and rotations (Bremmer et al., 2002). These areas in addition to the parieto-insular vestibular cortex (PIVC) demonstrate the cortical involvement in the processing of self-motion perception, heading and gaze control.

Most vestibular research focuses on the encoding of gravito-inertial signals and this is evident given the profound knowledge about the vestibular end organ, and its projections to brain stem and cerebellar areas. However, we think it will add to a more complete understanding of vestibular functions when including higher processing areas in future research. The use of neuroimaging will certainly contribute but it is noteworthy to point out these techniques are in essence correlational and do not permit drawing causal conclusions about the functions that are associated with a particular pattern of brain activation. In our view, the advancement of the field depends largely on the future use of innovative behavioral techniques, the use of which is absolutely necessary to tap into the mechanisms that underlie vestibular cortical processes.

PubMed and Web of Science were the main databases that we used in order to search the literature. The key word “vestibular” was used in combination with the search terms “cognition”, “mental rotation” “spatial”, “body representation”, “somatosensory”, “tactile”, “body schema” and “pain” to retrieve the relevant articles. The identified literature was then hand-searched, and we excluded articles focusing exclusively on physiological mechanisms, remotely related neurological disorders (e.g., Morbus Parkinson), animal research, and, for the most part, memory research. The latter has been addressed in several review papers already, and therefore became a field of research that receives considerable attention already (Smith et al., 2010; Smith and Zheng, 2013). The main focus of this review article lies on studies using a manipulation of the vestibular input (e.g., body motion, CVS, GVS). Furthermore, the references of the included articles were screened and new studies that cite the identified articles were taken into consideration. The remaining articles can be clustered in three research areas: *spatial cognition*, *body representation*, and *affective processes and disorders*. We will outline below the relevant state of research in each of these areas separately. In each section, we discuss in brief the most relevant issues tied to the respective research area, followed by a broader discussion of higher vestibular processing at the end of this article.

## Spatial cognition

The vestibular system has been identified as one of the most important sources of our sense of spatial orientation (see handbook chapter by Merfeld, 2012). The contribution of vestibular signals for various space-related cognitive functions becomes evident in patients suffering from vestibular loss. These patients show impairments in path integration, navigation and spatial memory (e.g., Brandt et al., 2005; Péruch et al., 2005; Guidetti et al., 2008). For example, patients with bilateral vestibular loss showed impaired performance in a virtual version of the Morris water maze (Schautzer et al., 2003; Brandt et al., 2005). Interestingly, these patients also showed decreased hippocampal volume (Brandt et al., 2005). In patients with unilateral vestibular loss, spatial memory deficits are subtle and they are not accompanied by a hippocampal atrophy (Hüfner et al., 2007, 2009). A substantial amount of research on the vestibular involvement in spatial memory has been gained from studies with vestibular-lesioned rodents. This research strongly supports the notion that vestibular input is required for navigation and spatial memory (e.g., Baek et al., 2010; Besnard et al., 2012; Zheng et al., 2012). While no effects on hippocampal volume were found (Zheng et al., 2012), vestibular lesions can disrupt hippocampal theta rhythm (Russell et al., 2006), eliminate directional sensitivity in hippocampal head direction cells (Stackman et al., 2002), and lead to neurochemical and neurophysiological changes (Zheng et al., 2001, 2013). See Smith et al. (2010) for a complete review on the implications of vestibular information in memory in humans and animals alike. In this context it is noteworthy that in healthy human subjects, vestibular stimulation was found to enhance spatial and non-spatial memory (Bächtold et al., 2001; Wilkinson et al., 2008).

Interestingly, the vestibular system is not only involved in the processing of motion in the physical world, it also seems to play an important role in building and maintaining a mental representation of the world. Even though we are bound to physical space, we are able to represent objects and movements mentally in order to optimally predict actions, react to events, and solve problems. A case in point is mental rotation. Shepard and Metzler (1971) have shown that people rotate a mental representation of an object in order to match it with another similar object. The response times increase with increasing angular disparity between two objects, and thus embody the characteristics of real rotations. It has been shown that the mechanisms that underlie mental rotation processes are not exclusively visual in nature but they also involve motor processes (e.g., Wraga et al., 2005, for a review see Kosslyn et al., 2001). Not only can we rotate in our mind visual representations of objects but also a representation of our own body or body parts. This is the case when one imagines what one would see from a different viewpoint. Viewer rotations operate on the representation of one’s own body and they can be performed independently of the actual state of the physical body. Vestibular areas process body information caused by real body movements (translations and rotations), and, given the large amount of cortical vestibular representations, it is conceivable that some of the areas are drawn upon in an off-line mode. This would imply that brain areas dedicated to the processing of real body motion are also in the service of mental imagery when whole-body motion is merely imagined but not executed. Interestingly, there is so far only one neuroimaging study testing vestibular imagery (zu Eulenburg et al., 2013a). They found activation in the inferior parietal lobules, and these activations were stronger in the left hemisphere. A left sided parietal activation is in line with several studies on imagined self-rotations (e.g., Just et al., 2001; Vingerhoets et al., 2001) even though the activation pattern reported by zu Eulenburg et al. (2013a) was more inferior in the parietal lobe. Zu Eulenburg et al. first exposed participants to yaw rotation in an upright position, and the participants later had to recall this sensation while lying supine in the scanner. Participants were not debriefed about the strategy they applied when they recalled previous vestibular sensations. Had the participants in the supine position reproduced in egocentric coordinates the yaw stimulation they experienced on the chair, it would have resulted in an imagined barbecue rotation, which implies a change in body position with respect to gravity. Changes in body position with respect to gravity can influence performance in mental imagery tasks (Mast et al., 2003). Clearly, behavioral techniques need to be refined in order to better assess what participants are doing when they recall from memory previously experienced vestibular sensations. As pointed out by zu Eulenburg et al. (2013a), they used a non-visual first-person strategy that differed from similar mental body transformation tasks, which require visual stimuli. Interestingly, no activation in many other areas belonging to the vestibular network (e.g., the human analog of the PIVC) has been revealed. Another neuroimaging study investigated imagined locomotion and the results show an overlap with real locomotion (la Fougère et al., 2010). However, imagined locomotion was associated with early visual activation and this suggests that participants used a visual rather than vestibular strategy. It is likely that participants imagined self-motion by means of visual flow, and, indeed, there was a deactivation of vestibular areas such as the inferior parietal lobule during imagery. Vestibular deactivation has been reported repeatedly during visually induced self-motion (e.g., Brandt et al., 2002). This discrepancy illustrates that subtle changes in how people perform mental imagery tasks can substantially change brain activation, and it will be a challenge for future research to narrow down the cognitive processes. For example, it needs to be controlled whether participants imagine as vividly as possible the experience of self-motion with or without the aid of imagined visual stimuli. A similar distinction is known for the assessment of motor imagery, for which a kinesthetic^1^ subscale and a visual subscale have been defined in the Vividness of Motor Imagery Questionnaire (VMIQ; Isaac et al., 1986). Inevitably, locomotion in real life stimulates the vestibular system, and if imagined locomotion reenacts the characteristics of real body motion we would expect involvement of vestibular areas so that kinesthetic imagery has more vivid perceptual qualities. However, it is possible that vestibular imagery may be inherently different from other types of sensory imagery such as visual or auditory. Therefore, it may be difficult for participants to perform vestibular imagery without relying on other sensory input (e.g., tactile or visual), which often accompanies the actual physical motion. However, vestibular stimulation can provoke a conscious experience of its own, and we are able to perceive self-motion despite the absence of concurrent extra-vestibular stimulation.

### Vestibular influences on spatial cognition

An example of how vestibular input can interact with spatial processing can be found in the well established subjective visual vertical task (SVV). A luminous line is set to the apparently vertical orientation, and it has been shown that the SVV depends on the body orientation of the observer (e.g., Tarnutzer et al., 2010). Interestingly, variation of task demands changes the contribution of the gravity-receptive signals (Bronstein, 1999). Clinical studies with vestibular patients suggest less impairment in the perception of body position (Bisdorff et al., 1996; Bringoux et al., 2002) or the adjustment of the gravity-referenced eye level (Bringoux et al., 2007) when compared to the typical pattern known from the assessment of the SVV. Spatial orientation tasks are an interesting front door for further exploring spatial cognition because performance in cognitive tasks also changes as a function of body position. For example, changing the inputs of the otoliths by means of the orientation of the observer has an impact on how objects are mentally manipulated (Corballis et al., 1976, 1978; Gaunet and Berthoz, 2000; Mast et al., 2003). In the same vein, specific changes in mental transformation tasks have been investigated through own-body rotation (van Elk and Blanke, 2013), changing the input from the semicircular canals by CVS (Mast et al., 2006; Falconer and Mast, 2012), changing the input of the vestibular nerve by GVS (Lenggenhager et al., 2008; Dilda et al., 2012), microgravity studies (Matsakis et al., 1993; Leone et al., 1995; Grabherr et al., 2007; Dalecki et al., 2013) and clinical studies with vestibular patients (Grabherr et al., 2011; Péruch et al., 2011; Candidi et al., 2013). It is important to point out that some studies provide evidence that vestibular input influences performance in mental rotation tasks only then when the task involves a representation of one’s own body or perspective (Lenggenhager et al., 2008; Dilda et al., 2012; Falconer and Mast, 2012). Congruent motion can facilitate mental rotation of one’s own body (Falconer and Mast, 2012; van Elk and Blanke, 2013) while absent or disturbed vestibular input can impair it (Grabherr et al., 2007; Lenggenhager et al., 2008). Some studies on vestibular patients show deficits in mental transformation of bodies and objects alike, suggesting that spatial-cognitive abilities are more globally impaired in these patients (Péruch et al., 2011; Candidi et al., 2013), whereas there is also evidence for a more pronounced impairment in mental rotation of bodies (Grabherr et al., 2011). These studies demonstrate that vestibular input or the lack thereof can interfere with or facilitate specific cognitive tasks. Even though the sum of these studies do not yet provide a fully coherent picture as to how vestibular processes are nested within spatial transformation abilities, they provide first evidence that the processing of vestibular information overlaps with specific cognitive spatial abilities. It will be interesting to investigate how the effects of body position on cognitive tasks relate to the spatial orientation tasks mentioned above, and whether the underlying gravity-receptive mechanisms differ between tasks.

The vestibular-cognitive interactions described above focused on cognitive processes that included mental spatial transformation of an object, body or body part. Beside this, vestibular input can also influence the processing of information that is represented in a specific spatial order. An illustrative example are numbers. In Western cultures, small numbers are cognitively represented in the left, and large numbers in the right side of space, following the concept of the “mental number line” (see Hubbard et al., 2005, for a review). The small-left and large-right-association also depends on vestibular information. For example, tuning the head to the left side leads to the production of smaller numbers than turning the head to the right side in a random number generation task (Loetscher et al., 2008). Similar effects have been found with horizontal passive whole-body motion in the dark, thus separating the vestibular input from neck afferences (Hartmann et al., 2012b; see also Hartmann et al., 2012a). A related concept of the mental number line is the mental time line, assuming that also temporal events are represented with a spatial entity. According to the concept of the mental time line, past events are associated with left or back space, and future events with right or front space (e.g., Arzy et al., 2009; Ulrich and Maienborn, 2010; Ulrich et al., 2012). In accordance with this, Miles et al. (2010) found that thinking about the past is accompanied by backward body sway, and thinking about the future by forward body sway. Similarly, it has been shown that future-related words were processed faster during forward when compared to backward passive whole-body motion, pointing again to vestibular contributions to these effects (Hartmann and Mast, 2012). A possible explanation for these findings could be the shift in spatial attention that is associated with the displacement of the body in space. Passive self-motion around the earth-vertical axis has been shown to shift spatial attention in the direction of motion: leftward motion facilitated the processing of a dot presented on the left side of the screen or a tactile stimulus applied on the left side of the body, whereas the opposite pattern was found for rightward motion (Figliozzi et al., 2005). Such a shift in spatial attention could also influence the processing of spatially represented stimuli. For example, moving leftwards makes small numbers more accessible because spatial attention is shifted to the left side of the mental number line.

### Cognitive influence on vestibular perception

Interestingly, there is also an effect in the reverse direction: cognitive processes can change the perception of vestibular stimuli. To date, it is still widely believed that self-motion perception is determined by characteristics of sensory receptors, and it has been shown that vection induces subtle sensorimotor changes in arm movement control (Bringoux et al., 2012). Interestingly, self-motion perception can be modulated by high-level visual input like seeing another person in motion (Lopez et al., 2013, see Deroualle and Lopez, 2014, this issue, for a review on vestibular information in social cognition). More surprisingly, Wertheim et al. (2001) showed that prior knowledge of motion can influence the perception of passive self-motion. Recently, Hartmann et al. (2012b) could demonstrate that processing small or larger numbers facilitated the detection of leftward or rightward rotation. Yet other studies have used mental imagery of visual motion, which also influences the perception of vestibular stimuli (Mast et al., 1999, 2001; Mertz and Lepecq, 2001). For example, mental imagery of self-motion by means of imagined vection stimuli can influence the perception of physical self-motion; recognition of target acceleration was improved when participants imagined themselves passively moving in the same direction, and, likewise, performance was impaired when the direction of imagined and perceived self-motion were incongruent (Mertz et al., 2000).

What needs to be more thoroughly investigated in future research is whether changes in thresholds have their origin at the level of sensitivity or at a later decision stage. Hartmann et al. (2013) could show that sensitivity in self-motion perception cannot be changed as a function of perceptual learning, and therefore, it is more likely that a bias rather than sensitivity is responsible for changes in thresholds. It is interesting that Rodionov et al. (2004) have shown that imagined body rotations can induce a typical nystagmus in healthy participants, and this findings suggests that imagined movements can penetrate the level of early oculomotor mechanisms. Gianna-Poulin et al. (2001), however, did not find a reduction of postrotatory nystagmus during imagined body tilts after prolonged yaw rotation. They conclude that velocity storage mechanisms are not malleable by cortical input. More research is needed to better investigate under which conditions and what kind of top-down processes can change oculomotor control. More specific knowledge about top-down influences on vestibular perception and vestibular reflexive responses may likely have a potential for application. For example, the use of cognitive procedures such as mental imagery training may be beneficial in vestibular rehabilitation (Lopez et al., 2011).

## Body representation

The sense we have of our own body, that we own it and have agency over it, that we can integrate our physical selves with our environment in functional ways, are sophisticated capacities that are often taken for granted. Head and Holmes (1911) proposed the terms body schema (related to proprioception and movement) and Schilder (1935) first coined the term body image (related to bodily awareness), and they described what our body feels like, and how we feel about our body. Ever since this terminology has been introduced there was ambiguity and confusion about what these capacities described. The current review relates to both proprioceptive capacity and bodily awareness. We will use the term *body representation* to collectively describe these capacities. Several clinical phenomena consistent with disruption of body representation were attenuated after CVS (for an overview on bodily disorders see de Vignemont, 2010, Table 1). These conditions include tactile extinction (Vallar et al., 1993; Kerkhoff et al., 2011; Schmidt et al., 2013), personal neglect (Cappa et al., 1987; Vallar et al., 1990), hemianesthesia (Vallar et al., 1990; Bottini et al., 2005), anosognosia (Cappa et al., 1987), somatoparaphrenia (Bisiach et al., 1991; Rode et al., 1992), macrosomatognosia (Rode et al., 2012) and phantom limb sensations (André et al., 2001; le Chapelain et al., 2001). Investigations into these phenomena have a long tradition, but only recently a few research groups began investigating potential effects of vestibular stimulation on tactile perception in healthy subjects. First, left cold CVS^2^ improved *tactile perception* in both hands (Ferrè et al., 2011, 2013a). Similar results were found when using left anodal and right cathodal GVS^3^—tactile perception was bilaterally enhanced—while right anodal and left cathodal GVS^4^ as well as sham stimulation had no effects (Ferrè et al., 2013b). Second, *tactile localization* was affected by GVS. Namely, tactile localization on the hand dorsum was shifted in the proximal–distal axis toward the wrist. This localization bias was greater after right anodal and left cathodal GVS compared to left anodal and right cathodal GVS (Ferrè et al., 2013c). Third, Lopez et al. (2012c) used left cold, right warm CVS to investigate the influence of vestibular input on the body schema. Participants performed a tactile distance comparison and stimuli applied to the hand were judged to be longer during CVS when compared to sham stimulation. In a second experiment they showed that the perceived size of the hand increased during CVS with respect to sham stimulation, implying an enlarged *somatorepresentation* due to vestibular stimulation. However, this is in contrast to Ferrè et al. (2013c) who found no effects of GVS on this type of task. Moreover, Lopez et al. (2010) used the “rubber hand illusion” paradigm and they found an increased illusory ownership for the left rubber hand and illusory location of touch when applying left anodal and right cathodal GVS. No effects were found when sham stimulation or right anodal and left cathodal GVS were used. Interestingly, no influences on the “objective” proprioceptive drift were observed (Lopez et al., 2010, 2012a). These studies extend earlier clinical reports about the association of vestibular dysfunction and abnormal bodily sensations that have been identified over a century ago (Bonnier, 2009).

Influences of vestibular stimulation on tactile perception and body representation in healthy subjects and patients alike seem plausible given the anatomical overlap of vestibular and somatosensory networks (Lopez and Blanke, 2011; Lopez et al., 2012b). A recent fMRI study that applied tactile and CVS in the same subjects revealed important overlapping cortical activation in the dorsal posterior insula and the parietal operculum (zu Eulenburg et al., 2013b). Moreover, an EEG study assessing somatosensory-evoked potentials elicited by electrical left median nerve stimulation before and after left cold CVS found a bilaterally enhanced N80 component, presumably generated in the parietal operculum (Ferrè et al., 2012). More research is needed to better control for the specificity of vestibular stimulation, and how long its effects persist over time.

Recently, Ferrè et al. (2013a) and Bottini et al. (2013, this issue) argue that CVS exerts specific effects that can be interpreted as sensory signal management. By modulating other sensory modalities vestibular signals can help to flexibly adjust to changing information with respect to the relation between the body and the external world, and therefore better anticipate future motor and perceptual events. According to the authors, CVS activates specific cortico-subcortical networks linking vestibular and somatosensory processes. We think that this connection has far reaching consequences beyond the somatotopic representation in the brain. As we will see below some studies suggest that vestibular information can alleviate pain, and we will also describe in more detail how it interfaces with affective processes.

### Vestibular stimulation and pain

Parents often comfort and sooth infants, when they just hurt themselves, by carrying and rocking them, thus providing plenty of vestibular stimulation (for scientific evidence for the soothing effect of rocking see e.g., Korner and Thoman, 1972; Byrne and Horowitz, 1981). The soothing effect may not be attributed to vestibular influences alone but so far—vestibular perception and pain—have rarely been combined in investigations although other sensory cues such as visual (Moseley et al., 2008a; Longo et al., 2009; Ramachandran et al., 2009) and tactile (Moseley, 2008; Moseley et al., 2008b; Moseley and Wiech, 2009) cues have been revealed to impact pain. The insular cortex is recognized as an important interoceptive center (Craig, 2009) and recent experiments suggest it might also provide a possible interaction between vestibular and nociceptive processing (zu Eulenburg et al., 2013b).

Pain is about protection of the body and chronic pain is associated with a range of disrupted bodily representations (for reviews see e.g., Moseley et al., 2012; Haggard et al., 2013). That vestibular stimulation attenuates similar disruptions raises the tantalizing possibility that it may also modulate pain. In fact, Harris hypothesized already in 1999 that CVS could relieve pain without accompanying tissue damage (e.g., phantom pain in an amputated limb). He hypothesized that pain arises as consequence of a mismatch between afferent sensory information and (motor) intention and that vestibular stimulation may help to restore balance in shared brain areas (Harris, 1999). Although there is limitated empirical support for the mismatch theory (e.g., Wand et al., 2014), there are a few studies reporting on the beneficial effect of vestibular stimulation. The first account that CVS can alleviate pain comes from participants with amputations and phantom limb perception (André et al., 2001). All of 10 amputees with painful phantom limb perception reported temporary relief after CVS (through the replacement with a normal non-painful phantom limb). Similar reports were made in two paraplegic patients with painful phantom limb perception (le Chapelain et al., 2001). The authors argue that vestibular information aids in reconstructing the global body schema (André et al., 2001), pinpointing the interaction between body representation and pain in these patients.

Shared brain structures for nociceptive and vestibular information processing have been proposed (for a review see Balaban, 2011). Recent clinical studies show yet again encouraging findings. Two patients with thalamic pain syndrome were suffering from severe pain in the contralateral body side after stroke for several years showing little relief from medication. Cold water CVS of the left and the right ear showed an important decrease in pain ratings, while sham stimulation had no effect. When the procedure was repeated just once, the positive effects lasted for several weeks (Ramachandran et al., 2007a,b). Nine patients with central post-stroke pain (CPSP; McGeoch et al., 2008) and one patient with transverse myelitis of the cervical spinal cord who developed right-sided central pain (McGeoch and Ramachandran, 2008) received treatment with a similar CVS protocol. Again, overall pain ratings decreased significantly. Interestingly, these patients reported that pain relief was greatest in the upper body (face and arm) while there was only little relief for the lower body (leg). It has been hypothesized that the posterior insula and/or anterior cingulate cortex (ACC) play a key role in the neural underpinnings of pain alleviation induced by CVS. Vestibular stimulation activates the posterior insula, which in turn is proposed to inhibit the generation of pain in the ACC (Miller and Ngo, 2007; Ramachandran et al., 2007b; McGeoch and Ramachandran, 2008). This seems to be supported by the findings of a single-case study using MEG measurements before and after CVS revealing reduced ACC activation. This patient with CPSP reported decreased pain up to 4 days after treatment (McGeoch et al., 2009). Furthermore, results from three patients suggest that CVS can reduce pain only when the main vestibular cortical areas are spared (McGeoch et al., 2008; Rode et al., 2012). Given the low sample size in the studies reported above caution needs to be taken and the influence of potential biases will need to be controlled for in future research. In this context it is of interest that neurotransmitters can possibly help explain the beneficial effects of vestibular stimulation. Indeed, GVS was found to increase the release of GABA in rats (Samoudi et al., 2012) and gabapentin—a GABA analog—is a frequently prescribed drug to treat pain (e.g., neuropathic pain, Jensen et al., 2009).

Surprisingly, and to the best of our knowledge, there is only one study that has investigated whether vestibular stimulation can affect pain in healthy participants. Ferrè et al. (2013a) assessed tactile and pain thresholds in healthy participants before and after left-sided cold CVS. They showed that while tactile thresholds decrease, pain thresholds increased after stimulation, but no control condition was used, and the lack of a control intervention makes it hard to exclude diffuse noxious inhibitory control.

In sum, while these results are encouraging, more research with sound methodology (e.g., well-controlled studies that include sham conditions, randomized control studies) and greater sample size is needed. At this point it is still difficult to draw a conclusion whether the influences observed underlie a direct function or whether they are rather unspecific. Conceivable confounders may include stress (Saman et al., 2012), attentional or placebo effects. Moreover, future clinical studies need to clarify which chronic pain conditions can benefit from vestibular stimulation and which cannot. Moreover, which is the best vestibular stimulation protocol for which type of pain and what are the underlying neural underpinnings. While common vestibular and pain processing areas have been outlined, imaging studies yet need to assess possible structural changes due to vestibular stimulation. We hypothesize that vestibular stimulation can alleviate pain by contributing to ameliorate the impaired body schema and help restore the “body matrix” (Moseley et al., 2012). Thus, conditions like complex regional pain syndrome or chronic back pain, where disturbed body representations have been described (e.g., Moseley, 2005, 2008; Moseley et al., 2008a,b), should benefit the most. We even go on to speculate that other clinical conditions that involve disturbances of body representation such as Anorexia Nervosa (e.g., Garfinkel et al., 1978; Keizer et al., 2013) may profit as well. While Anorexia Nervosa has not yet been experimentally assessed and linked to vestibular stimulation, other mental disorders—outlined below—have been investigated. However, on this note, it is important to point out that a recent study that assessed the effect of CVS in participants suffering from body identity integrity disorder (BIID) was not able to significantly reduce the estrangement of the affected limb(s) (Lenggenhager et al., 2014). Against the background of publication bias, negative findings are informative and clearly indicate the need for well-controlled studies with sufficient sample size.

## Affective processes and disorders

Links between vestibular dysfunction, anxiety and panic disorders have been suggested for a long time (for an overview see Balaban and Jacob, 2001). Patients with a vestibular disorder have a higher probability of suffering from depression, anxiety and panic disorders (with and without agoraphobia) (Eagger et al., 1992; Godemann et al., 2004; Guidetti et al., 2008; Gazzola et al., 2009). Likewise, patients with a psychiatric disorder (such as anxiety or panic disorder) often report vestibular symptoms such as dizziness. A current model that explains the high comorbidity between psychiatric symptoms in vestibular disorders proposes that depression and anxiety are a reaction to the distress of living with vestibular dysfunctions (Jacob et al., 1996). This explanation does not provide any insights into the underlying mechanisms. Several studies concluded that patients with agoraphobic, panic or motion discomfort disorder have problems with multisensory integration (Yardley et al., 1994; Jacob et al., 1996, 1997). Vestibular patients rely predominantly on visual (Dieterich et al., 2007) and proprioceptive cues (Bles et al., 1984). Inadequate integration of visual and proprioceptive information may result in the fear of falling (*space and motion discomfort*) (Jacob et al., 1996). Interestingly, Viaud-Delmon et al. (2002) could show that participants with high trait anxiety also rely more on external visual cues in a visual-vestibular conflict task. The authors proposed that vestibular dysfunctions in high trait anxious participants may not affect low-level vestibular functions but rather involve problems with higher order cognitive integration of self-motion signals contributing to spatial cognition (Berthoz and Viaud-Delmon, 1999). However, previous studies show that low-level vestibular functions are also affected. Yardley et al. (1992) found an increased slow phase velocity of the VOR in high state anxious participants, which is an indicator for risk of dizziness. Studies in panic disorder patients have shown abnormal responses in postural stability and abnormal oculomotor functioning (Sklare et al., 1990; Redfern et al., 2007).

Taken together, epidemiological studies support a high comorbidity between vestibular and emotional disorders. However, the underlying neural mechanisms remain unclear. Although the limbic and prefrontal contributions to the processing of emotional information are well established, the influence of vestibular processes is not yet thoroughly understood. Recent evidence suggests that the interaction between vestibular functioning and emotion processing may underlie shared subcomponents of a common neural network (Preuss et al., 2014a).

### Brainstem links and cortical connections

Several brain regions are commonly involved in vestibular and emotion processing (Yardley et al., 1999; Balaban and Jacob, 2001). Widespread vestibular-cortical projections may account for the comorbidity between the vestibular and psychiatric symptoms (Balaban et al., 2011; Lopez and Blanke, 2011; Gurvich et al., 2013). One key anatomical region in the brainstem is the parabrachial nucleus (PBN; Balaban and Thayer, 2001; Balaban, 2004). The PBN provides a direct link between the vestibular system and emotion processing, and it has bi-directional connections to the vestibular nuclei (Balaban, 2002, 2004; Balaban et al., 2011). Furthermore, the PBN network exerts reciprocal connections to the amygdala, hypothalamus, locus coeruleus and prefrontal cortex, which are all areas of the limbic system and have been associated with affective disorders (Schuerger and Balaban, 1999; Gorman et al., 2000; Balaban and Thayer, 2001). Yet other brainstem regions that have reciprocal connections with the vestibular nuclei are the raphe nuclei and the locus coeruleus. Both regions are involved in psychiatric conditions. The raphe nuclei send serotonergic and nonserotonergic projections to the vestibular nuclei (Kalén et al., 1985; Halberstadt and Balaban, 2006) and axon correlates to the central amygdaloid nucleus, suggesting a modulating effect of vestibular pathways on affective control (Halberstadt and Balaban, 2006). The locus coeruleus sends noradrenergic projections to the vestibular nuclei (Schuerger and Balaban, 1999) and also responds to vestibular stimulation (Manzoni et al., 1989).

Within the vestibular network, the ACC has been considered as the bridge between vestibular-sensorimotor areas and the affect divisions of the prefrontal cortex that entail motivational states (Bush et al., 2000). The insular cortex is the core region that receives input from the vestibular nuclei in the brain stem (Akbarian et al., 1994). The prefrontal cortex plays a more indirect role in the processing of vestibular information and suppresses the impact of irrelevant sensory information and regulates incoming sensory stimulation that can overwhelm cognitive capacities (see for example Yamaguchi and Knight, 1990, 1991; Rule et al., 2002; Angrilli et al., 2008). For example, coherent full-field visual motion stimuli elicit the sensation of self-motion such as the illusory feeling of motion when sitting in the stationary train while it is only the neighboring train that pulls out of the station in the direction opposite to the felt motion. However, people react differently to full-field motion stimuli, and in laboratory experiments additional tasks can weaken the strength of vection (Seno et al., 2011). Prefrontal regions may exert their influence over the vestibular cortical regions through its indirect connection with the premotor areas and the temporal lobes (Damasio and Anderson, 2003). However, the prefrontal cortex does not only regulate incoming sensory information but it also serves the regulation of emotions. Indeed, Carmona et al. (2009) proposed a conceptual model, integrating both mechanisms and the role of vestibular information. The first mechanism (“sensorimotor-affective mechanism”) involves the dense interconnection of the vestibular sensory areas and the anterior cingulate gyrus and the prefrontal cortex. Frontal regions (via motor areas and anterior cingulate gyrus) exert a regulatory influence on vestibular areas and attenuate sensory stimulation as described in the example given above (Carmona et al., 2009, see also Akbarian et al., 1994; Nishiike et al., 2000). The second mechanism (“autonomic-affective mechanism”) involves the direct link between the vestibular nuclei with limbic structures. Carmona et al. (2009) propose that the frontal lobes exert an inhibitory control over the vestibular nuclei in the brainstem. As a consequence, intense negative stressors (triggered though vestibular dysfunctions, dizziness, disorientation, motion sickness etc.) burden frontal resources, resulting in diminished capacity to allocate resources for attenuating activation in posterior sensory and autonomic regions (e.g., the insula and the temporal lobe). This leads to difficulties in maintaining sensorimotor coordination in balance and regulating arousal.

### Vestibular stimulation, affect and laterality

Recent evidence suggests that vestibular stimulation can have a positive effect on affect and psychiatric disorders. Dodson (2004) showed in a single case study that left cold CVS exerts a beneficial effect on manic symptom severity. The positive effect of CVS on mania can be attributed to an activation of right hemispheric structures. Levine et al. (2012) showed an increase in bilateral frontal and central alpha EEG band activation in three patients suffering from a schizoaffective disorder. They proposed that left cold CVS can have a short lived beneficial effect on manic delusions and insight of illness that appear in mania and other types of psychoses (i.e., schizophrenia).

Hemispheric laterality in the processing of emotions and affective information has been proposed for many years. Lateralization of vestibular processes, however, is relatively new and has come to light in the last years. Dieterich et al. (2003) could show a dominance of vestibular cortical function in the non-dominant hemisphere. Several studies reported greater right than left hemispheric activation in the vestibular cortex (Bottini et al., 1994, 2001; Dieterich et al., 1998, 2003; Lobel et al., 1998; Fasold et al., 2002; Kahane et al., 2003; Schlindwein et al., 2008). Models of hemispheric lateralization of emotions have proposed that the hemispheres process emotional information differently (“valence-specific hypothesis”) (Tucker, 1981; Davidson and Fox, 1982; Davidson, 1992).

Although conversely discussed, the valence-specific hypothesis assumes that negative emotions are processed in the right hemisphere whereas positive emotions are processed in the left hemisphere. Pettigrew and Miller (1998) proposed a model for bipolar disorder (“sticky switch model”), which views mania as the endpoint of relative left hemispheric activation and depression as the endpoint of relative right hemispheric activation. In order to examine this hypothesis, Miller et al. (2000, 2003) used a binocular rivalry task in which two competing images are presented to each eye separately. This leads to an alternating perception of each image mediated by alternating hemispheric activation (*interhemispheric switching*). The author reported a slower alternation rate in patients with bipolar disorder leading to the conclusion of a slow *interhemispheric switching* in these patients—the brain becomes “stuck” in one of two states (Miller et al., 2003). Interestingly, CVS can be used to effectively modulate the alternation rate in binocular rivalry (Miller et al., 2000).

The findings of these studies all lead to the conclusion that vestibular stimulation could be a potential therapeutic treatment in mania and depression. Left cold ear CVS, through its activation of the right hemisphere, was proposed to reduce manic symptom severity whereas right cold ear CVS, through its activation of the left hemisphere, was proposed to reduce symptoms in depression. Since then, clinical evidence has indeed shown a beneficial effect of left cold CVS on manic symptom severity (Dodson, 2004; Levine et al., 2012). So far, only one study investigated the effect of CVS in major depression (Ried and Aviles, 2007). Patients suffering from a major depressive disorder show a bilateral decrease in slow phase velocity of the nystagmus following 30°C and 44°C CVS. Unfortunately, changes in mood were not assessed. In addition to clinical findings, a recent study could show that CVS has mood and affect regulating effects in healthy participants (Preuss et al., 2014a). Preuss et al., 2014b could show that left cold CVS decreases affective control whereas right cold CVS increased affective control for positive stimuli in an affective Go/NoGo task. The authors concluded that the change in affective control is due to an activation of the underlying emotional network. Furthermore, a recent study could show that CVS can also influence decision outcome in purchase decision-making. Left cold CVS decreased the probability of purchasing a product and also desirability of the products (Preuss et al., 2014b). McKay et al. (2013) could show that cold CVS of the left ear decreases unrealistic optimism in healthy subjects. Unrealistic optimism is a bias that causes a person to believe that he or she is less at risk of experiencing negative events (such as illnesses). Hence, unrealistic optimism represents some sort of delusion or subclinical anosognosia in healthy subjects. Indeed, the authors conclude that both unrealistic optimism and anosognosia (see also Cappa et al., 1987) share the same underlying mechanisms. Future studies should thus further investigate the effect of CVS in affective disorders as CVS might regulate mood and affect in major depression and mania but also give insight into delusion and illness denial. Vestibular stimulation by non-invasive methods, and its potential use in the treatment of the conditions outlined above is by far not exploited.

## Discussion

Activation of the vestibular system leads to widespread activity in different cortical areas but the extent of all the functions the vestibular network is involved in are not yet clear. We have described three streams of research, which have in common that they can be modulated by vestibular information. None of these areas belongs to traditional domains of vestibular research like postural control, VOR, and self-motion perception. Therefore, the question about the purpose of vestibular information in these tasks is by all means justified. Before searching for answers to this question, one needs to take a step back and wonder why the answers are not yet known already. Several conceivable explanations can be brought forward. First, it is clear that the paucity of knowledge in a given field is proportional to the amount of research carried out. The number of 24,163 articles shows up in PubMed with the term “vestibular” in the title or abstract. In comparison, there are 261,164 with the term “visual” (PubMed, October 18th, 2013, the results are corrected for contributions with both terms). This means that there are 10.8 times more articles published in the visual domain. Indeed, the visual system processes a wider range of different stimuli such as motion, color, orientation, form and so forth, and to date, the knowledge of cortical neurophysiology is by far better understood when compared to the vestibular domain. It is a relatively recent trend that vestibular information has gained some more attention based on growing evidence of its involvement in tasks that are seemingly remote from known vestibular functions. Second, the lack of knowledge is—at least in part—caused by the experimental effort that is necessary to carry out vestibular experiments. For example, motion devices that are capable of providing well-controlled vestibular stimuli do not belong to the standard equipment of perception research laboratories. They are expensive and often require labor intense software development for machine control. Moreover, true motion cannot be applied in combination with most neuroimaging devices, and the use of techniques like CVS and GVS entail problems of inducing sensory conflicts (e.g., during CVS participants are in a pitched-back position and receive a stimulation of the canals while the otoliths signal no change in posture). A promising new technique for vestibular research is functional near-infrared spectroscopy (fNIRS) because participants can be tested in different body positions as compared to fMRI studies, which only allow measurements in the supine position. A recent fNIRS study by Karim et al. (2013) used CVS and confirmed previous fMRI and PET activation and lateralization patterns on the cortical surface. Last but not least, it is puzzling that sporadic research findings in vestibular science are not followed up more rigorously. A case in point is the work by Sauvan and Peterhans (1995) and Sauvan (1999). They have shown that a proportion of neurons in occipital area V2 shift their orientation specificity from retinal to gravity related coordinates during static roll tilts. This finding brings up fundamental questions about multisensory integration and the level at which otolith information is fed into the visual system to achieve space constancy. The striking phenomenon of a perceptually upright world when the head is tilted is not understood, but, currently, there seems to be relatively little interest in a thorough investigation of this fundamental spatial ability, also among the large community of vision scientists. To our knowledge, no follow-up study has been conducted since Peterhans and Sauvan’s work, and only few articles made reference to their work.

What are the functions vestibular information can have in tasks that are seemingly remote from known functions like self-motion perception or postural control? First of all, the vestibular system establishes a frame of reference for spatial orientation. It provides information about the spatial relation of the self with respect to the world. Bringing the gravito-inertial force (GIF) vector in line with the body’s main axis is the set value in any type of behavior. The GIF is an allocentric cue and its perception connects egocentric body-based coordinates with external world coordinates. Sudden and unintended deviations from the GIF are rapidly compensated for by postural adjustments. In case of obstacles a continued and undesired deviation from the GIF indicates vulnerability and therefore distress, which is a warning signal and thus reinforces the motivation to initiate a change in posture. Disequilibrium is a stressor indicating an unintended mismatch between frames of reference. This is where the interface between balance and affective processes—both phylogenetically old mechanisms—comes into play. Affective processes signal importance, and trigger a more or less elaborated coping behavior, depending on the organism’s capacities. In this sense, postural reflexes involve more than just a change in body posture. Disequilibrium and falls are a threat to the organism that triggers an affective response. In real life, it is possible that the body’s immediate and fast reflex loops precede the affective response such as when we miss a step on the stairs without actually falling. We start to feel the increase in heart beat just after having successfully avoided a fall. The existence of this vestibulo-affective interaction will unfold to its maximum when immediate correction of posture is impeded. Affect and body motion are interconnected, and future research will be needed to explore the underlying mechanisms. Moreover, a bodily representation of the self, for example, serves as a reliable tool for mentally simulating future events without having to act them out. This representation can trigger affective responses because the representation constitutes the self. Vividly imagining a fall can cause fear and affective responses, just as in the real world walking down a steep slope on a frozen street inevitably causes fear of falling. Mental imagery does not only draw in an off-line mode on sensory representations but it also involves affective processes depending on the relevance of the imagined content.

This review highlighted the role of vestibular information beyond guiding the body through the physical world. Cognitive processes such as mental spatial transformations or the processing of spatially represented information is nested with the processing of vestibular information. These vestibular interactions with higher-order spatial cognition could be based on the reference frame that the vestibular system provides the brain with. The otolith organs constantly update our body position in space with respect to gravity and this allows for representing the outside world within defined spatial vectors (horizontal, vertical, and transversal axis). The same spatial reference system manifests itself in cognitive tasks. According to the grounded cognition theory, space as a more concrete domain is used to structure, represent, and understand abstract concepts such as number magnitudes or temporal events that cannot be experienced directly through our senses (e.g., Lakoff and Johnson, 1980; Boroditsky, 2000; Gallese and Lakoff, 2005; Barsalou, 2008).

The vestibular system is a phylogenetically old structure that is nested and intertwined with subcortical and cortical structures. On the one hand, some reflex arcs can be considered vestigial such as ocular counterrolling because it does not fully counteract the impact of head tilts on the retinal image. On the other hand, however, the same efferent signal driving ocular counterrolling may be involved in other processes such as determining the gain of the visual field influence on the perception of the visual vertical as proposed by Bischof (1974). The multiple connections with other systems make it extremely complex to tease apart when and how vestibular input affects the processing of other modalities or affective responses. It is interesting that the vestibular system alone might be less plastic when compared to other sensory systems (Hartmann et al., 2013). Merfeld (2012) pointed out that vestibular organs appeared very early in evolutionary history and have remained relatively unchanged. It is therefore conceivable that an increase in vestibular sensitivity as a result of perceptual learning could have negative side effects such as motion sickness or an exaggerated response to small accelerations, for example during transport, and changes in sensitivity might affect all other modalities that are connected to the vestibular system. In fact, there is evidence that the vestibular system has numerous connections and overlaps with other functional networks. Based on recent research in spatial cognition, body representation and affective processes we presented an overview that will hopefully stimulate and contribute to future research in vestibular science.

## Conflict of interest statement

The authors declare that the research was conducted in the absence of any commercial or financial relationships that could be construed as a potential conflict of interest.

## Footnotes

^1^The VMIQ-2 is a revised version of the original VMIQ (Roberts et al., 2008). It makes a more precise distinction between internal imagery (participants visually imagine performing an action) and kinesthetic imagery (participants imagine themselves performing an action).

^2^Known to activate predominantly the right hemisphere (Miller and Ngo, 2007).

^3^Known to activate predominantly the right hemisphere (Utz et al., 2010).

^4^Known to bilaterally activate vestibular cortical areas (Utz et al., 2010).
